# Resilience and response of marine microbes to the future ocean and a marine heatwave — insights from a mesocosm experiment

**DOI:** 10.1093/femsec/fiag042

**Published:** 2026-04-22

**Authors:** Jan D Brüwer, Micah Reismann, Antje Wichels, Uwe John, Josefin Schmidt, Cédric L Meunier, Bernhard M Fuchs, Inga V Kirstein

**Affiliations:** Max Planck Institute for Marine Microbiology, 28359 Bremen, Germany; Max Planck Institute for Marine Microbiology, 28359 Bremen, Germany; Institute for Biosciences, University of Rostock, 18059 Rostock, Germany; Alfred-Wegener-Institut, Helmholtz-Zentrum für Polar- und Meeresforschung, Biologische Anstalt Helgoland, 27498 Helgoland, Germany; Alfred-Wegener-Institut, Helmholtz-Zentrum für Polar- und Meeresforschung, Biologische Anstalt Helgoland, 27498 Helgoland, Germany; Alfred-Wegener-Institut, Helmholtz-Zentrum für Polar- und Meeresforschung, Biologische Anstalt Helgoland, 27498 Helgoland, Germany; Alfred-Wegener-Institut, Helmholtz-Zentrum für Polar- und Meeresforschung, Biologische Anstalt Helgoland, 27498 Helgoland, Germany; Max Planck Institute for Marine Microbiology, 28359 Bremen, Germany; Alfred-Wegener-Institut, Helmholtz-Zentrum für Polar- und Meeresforschung, Biologische Anstalt Helgoland, 27498 Helgoland, Germany

**Keywords:** 16S rRNA gene sequencing, fluorescence in situ hybridization, image analysis, cell division, mortality, virus like particles, filamentous bacteria

## Abstract

Anthropogenic influences are reshaping ocean conditions, with rising sea surface temperatures, elevated CO_2_ concentrations, and shifting nutrient dynamics occurring alongside more frequent extreme weather events. While previous studies indicated that various planktonic taxa may be impacted by long-term environmental change, the response of the microbial community remains less explored, and we know particularly little about how short-term events such as marine heatwaves interact with long-term environmental changes. Here, we investigated the impact of a heatwave (+2°C for 5 days) on the microbial community in mesocosms mimicking ambient and future coastal conditions (1000 ppm CO_2_, +3°C, Nitrogen: Phosphate ratio of 25), according to IPCC predictions (high-emission scenario RCP 8.5) for 2100. We used 16S rRNA gene sequencing, fluorescence microscopy, and experimental approaches, to study the microbial community taxonomic composition, cell abundances, virus-like particle abundances, as well as cell division and mortality rates. Our results indicate that future coastal conditions may impact the microbial community composition, biodiversity, as well as the abundance of total bacterioplankton. In contrast, the marine heatwave we simulated had a smaller impact on the microbial community composition, and we did not observe any effect of the heatwave on virus-like particle counts and cell division rates. In conclusion, our findings suggest a certain resilience of the microbial community to short-term thermal disturbances.

## Teaser

The microbial community is mostly resilient to a short-term marine heatwave.

## Introduction

Anthropogenic activities and the resulting global climate change are altering environmental conditions, especially in coastal systems (Pörtner et al. 2022). Under the Representative Concentration Pathways (RCP) 8.5 scenario, atmospheric CO_2_ concentrations are projected to reach 1000 ppm by 2100, leading to a pH decline of approximately 0.3 units (Pörtner et al. 2022). The ocean has already absorbed about a quarter of total anthropogenic CO_2_ emissions (DeVries et al. 2017) and nearly 90% of the heat associated with climate change (Gattuso et al. 2015, Cheng et al. 2017), contributing to rising sea-surface temperatures. Coastal regions appear to experience these changes even more acutely (de Amorim et al. 2023). In the last six decades, sea-surface temperatures in German North Sea coastal waters increased ca. 60% faster compared to the North Atlantic ocean (40°N–60°N) (de Amorim et al. 2023). Furthermore, North Sea coastal waters are subjected to changes in dissolved nutrient concentrations with a steadily increasing nitrogen-to-phosphorus (N : P) ratio over the past decades (Van Beusekom et al. 2019, Balkoni et al. 2023). Together, multiple anthropogenic-driven changes (e.g. temperature, pH, and nutrient ratio) have been found to impact marine organisms, including phytoplankton at the basis of the marine food web. Elevated temperatures are expected to favor smaller-sized planktonic organisms (Gao et al. 2018, Moreno et al. 2022, Ahme et al. 2025) and heterotrophic processes over autotrophy (Chen et al. 2012, Boscolo-Galazzo et al. 2018, Ahme et al. 2025). Additionally, ocean acidification may enhance photosynthesis and biomass production (Gattuso et al. 2015, Moreno et al. 2022), including the release of carbon-rich compounds (Gao et al. 2018). While the effects of anthropogenic change (especially increased temperature and decreased pH) on the phytoplankton community have been thoroughly studied, much less is known about the microbial community of heterotrophic bacteria, which remineralize phytoplankton-derived substrates (Buchan et al. 2014).

Generally speaking, warmer temperatures are expected to increase microbial growth rates across taxa (Hutchins and Fu 2017, Berner et al. 2018, Barbosa et al. 2023). Heterotrophic bacteria often have thermal optima higher than *in situ* temperatures, though this offset between growth optima and *in situ* temperature varies seasonally (Joint and Smale 2017). For example, microbial communities in the English Channel exhibit an offset of 3°C difference in summer and 20°C in winter with all intermediates in the respective seasons (Joint and Smale 2017). At the long-term ecological research site Helgoland Roads in the German North Sea, it has been shown that changes in water current patterns leading to the inflow of nutrient-rich coastal waters enhance phytoplankton blooms. The subsequent short-term successions of bacterioplankton in response to these blooms are indirectly influenced by temperature, which serves as a niche-defining factor in the German Bight (Teeling et al. 2012, Lucas et al. 2015). Hence, the effects of temperature vary between taxonomic groups and may be region-specific. Warmer temperature are generally considered to favor smaller bacterial taxa, such as SAR11 (*Alphaproteobacteria*) (Joint and Smale 2017). However, a different study from the Baltic Sea reports *Alphaproteobacteria* (mainly SAR11) and *Gammaproteobacteria* benefitting from colder temperatures and *Bacteroidota* benefitting from warmer temperatures (Berner et al. 2018). In contrast to temperature, heterotrophic bacteria appear relatively resilient to ocean acidification. Most mesocosm studies from diverse regions, including the Baltic Sea (Bergen et al. 2016), the coast of Portugal (Barbosa et al. 2023), and New Zealand (Deans 2022), have reported no significant effects of increased CO_2_ concentrations on microbial cell abundances or community composition.

In addition to long-term effects of anthropogenic induced environmental changes, the frequency and severity of extreme events, such as marine heatwaves (MHWs), are increasing (Lee et al. 2023, Giménez et al. 2024). MHWs are defined as events lasting at least five days, during which sea surface temperatures exceed the 90^th^ percentile of the average seasonal climatology (Hobday et al. 2016). Despite their increasing impact and importance, the effects of short-term MHWs on microbial communities remain poorly understood.

Previous studies assessing the effects of large-scale MHWs and those lasting multiple weeks reported community shifts, including a shift to smaller heterotrophic bacteria (Brown et al. 2024), as well as the spread of *Vibrio* and *Planctomycetes* in coastal waters (Doni et al. 2023) and during the Blob in the North Pacific (Traving et al. 2021). However, information about cell division rates and mortality rates during MHWs, as well as the effect of MHWs during future coastal conditions remains largely unexplored.

To gain a more comprehensive understanding of the multifaceted impacts of climate change and extreme weather events on the marine microbial community, we conducted an integrated multiple driver mesocosm experiment, simulating both future coastal conditions and marine heatwaves. To simulate future environmental conditions, we simultaneously manipulated seawater temperature and pH, based on IPCC predictions for 2100, as well as dissolved N: P ratios based on the conditions expected in European coastal zones (Van Beusekom et al. 2019, Balkoni et al. 2023). Using 16S rRNA gene sequencing and fluorescence microscopy approaches (including fluorescence *in situ* hybridization (FISH)) we assessed the combined effects of current and future MHWs on microbial community abundance, diversity and composition. To gain deeper insights into the impact of a MHW on microbial communities we conducted additional experiments and analyses to study the effect of MHWs on cell division and mortality rates, as well as virus-like particle abundances in ambient conditions. Our study aims to evaluate the combined and individual effects of global change and MHWs on microbial diversity, community composition, and taxon-specific responses.

## Material and methods

### Mesocosm set-up

To assess the impact of MHWs on ambient and future coastal scenarios, an integrated multiple-driver experiment was conducted using a total of 16 mesocosms. The mesocosm design and set-up are described in detail in Meunier et al. (2025). Briefly, mesocosms were exposed to four scenarios with four replicates each. The scenarios contained ambient “AMB” conditions (ambient temperature, pH, pCO_2_, N: P ratio 16) or a future coastal scenario “ERCP” based on the RCP 8.5 scenario developed by the IPCC for the year 2100, extended with an additional adjusted nitrogen to phosphorous (N: P) ratio (+3.0 °C, -0.3 pH, pCO_2_ = 1000 ppm, N: P ratio 25). Additionally, each treatment was exposed to a moderate heatwave of +2°C for 5 days (“AMB+HW”, “ERCP+HW”) or not (“AMB”, “ERCP”) on day 11. Before and after the heatwaves, the temperatures were adjusted stepwise by 1°C d^-1^ for 2 days to reach the heatwave or ambient temperatures, respectively.

The mesocosms were located at the Wadden Sea Station, Alfred-Wegener Institute for Polar and Marine Research, Germany (Dummermuth et al. 2023). Each mesocosm (1800 l) contained an LDPE transparent bag (520 l) comprising the experimental water mass. The surrounding water was used for temperature regulation. The bags were filled with 1000 µm-filtered seawater from Sylt Roads Station 1 (55°1′48″ N, 8°27′36″ E) and were incubated between 1^st^ September and 27^th^ September 2021. The mesocosms were covered with an HDPE translucent lid, allowing 90% of photosynthetically active radiation to pass through. Additionally, the lid allowed for changes in pCO_2_ in the mesocosms to mimic future coastal scenarios. Mesocosms were mixed with a custom-build propeller attached to a mortar mixer (TC-MX 1400–2 E, Einhell Germany AG, Landau/Isar, Germany) at 50 r/m with a 1-min-mixing/30-min-pause interval.

### Measurements throughout the experiment

Abiotic parameters, including temperature, pH, total alkalinity, dissolved nitrogen, particulate carbon, nitrogen, and phosphate, were measured daily and are reported elsewhere (Meunier et al. 2025). Chlorophyll *a* concentrations were determined daily using spectral fluorometry at a fluorescence of 685 nm (AlgaeLabAnalyser, bbe Moldaenke GmbH, Schwentinental, Germany).

To study the microbial community of the mesocosm, 10 ml samples were fixed with 0.2 µm-filtered formaldehyde (1% final concentration) and immobilized on 0.2 µm pore size polycarbonate filters (Merck Millipore, Burlington Massachusetts, US). Filters were stored at −20 °C until further processing. An aliquot of 1 ml of the fixed 0.2 µm filtrate was immobilized on a 0.02 µm pore zise aluminum oxide filter (Anodisk Whatman, Maidstone, United Kingdom) to study the viral community by quantifying virus-like particle concentrations.

### Dilution grazing experiments

We further studied microbial mortality factors with a focus on ambient treatments, due to logistical reasons. As MHWs are increasing in occurrence, we wanted to understand their effect on mortality factors under the ambient conditions.

We conducted four dilution experiments (day 6, 13, 20, and 27) of the AMB and AMB+HW treatments to determine the impact of a MHW on cell division and grazing rates of the prokaryotic community after Landry and Hassett (1982). To exclude mesozooplankton and larger grazers, samples were sieved (200 µm) before dilution with 0.2 µm sterile-filtered seawater. Dilution series of 100% (undiluted), 50%, 30%, and 15% were prepared in 1 L cell culture flasks (Greiner, Kremsmünster, Austria).

The flasks were placed on a plankton wheel (ca. 1.2 r/m) to prevent sedimentation of the planktonic organisms and incubated with a day-to-night regime of 16–8 h (20–30 µmol photons m^−2^ s^−1^) for 24 h in a temperature-controlled room set at the *in situ* sea surface temperature of the corresponding day. After the incubation, samples were fixed and filtered for microscopy assessments (described below). Taxon-specific cell division rates were calculated according to Landry and Hasset (1982). In brief, net growth can be calculated with incubation time *t*, abundance at the start (*N_0_*) and at time-point *t* (*N_t_*), and the respective dilution factor d: Net growth = (1/t) * ln (N_t_/N_0_*d). The slope of the linear model of the apparent growth over the dilution factor is the grazing rate, and the *y*-axis intercept the cell division rate.

### DAPI, SYBR-gold, and fluorescence *in situ* hybridization (FISH) staining to count microbes and virus-like particles

To determine total cell counts, a subsample of the 0.2 µm polycarbonate filters was embedded in the anti-bleaching agent citifluor: vectashield (Citifluor Ltd, London, UK; Vector Laboratories, Burlingame, CA, USA), containing 1 µg ml^−1^ 4′,6-diamidino-2-phenylindole (DAPI), for microscopy and counting (see below). The 0.02 µm aluminum oxide filters for virus-like particle counts were cut into quarters. Each subsample was embedded in 0.2 µm-filtered 1 : 1 (vol: vol) PBS: glycerol, containing 0.01% (wt/vol; final concentration) p-phenylendiamine and 10x SYBR-gold (final concentration, Invitrogen, Waltham, Massachusetts, United Kingdom).

We used catalyzed reporter deposition—fluorescence *in situ* hybridization (CARD-FISH) to visualize and count individual taxonomic groups, following Fuchs et al. (2007). Briefly, a subsample of the 0.2 µm polycarbonate filters was first embedded in 0.1% LE agarose to prevent cell loss during sample handling. Cell walls were permeabilized using 10 mg ml^−1^ lysozyme in buffer (0.05 M EDTA, 0.1 M Tris-HCl) for 1 h at 37°C and 60 U ml^−1^ achromopeptidase in buffer (0.01 M NaCl, 0.01 M Tris-HCl) for 30 min at 37°C. Endogenous peroxidases were inactivated using 0.15% H_2_O_2_ in methanol for 15–20 min. Hybridizations were conducted in humidity chambers, containing specific formamide and NaCl concentrations (Table S1). Samples were incubated in hybridization buffer (900 mM NaCl, 20 mM Tris-HCl, 1% blocking reagent, 0.1 g l^−1^ dextran sulfate, 0.02% SDS, and varying formamide; Table S1) for 3 h at 46°C and subsequently washed in washing buffer (20 mM Tris-HCl, 5 mM EDTA, 0.01% SDS, NaCl concentration depending on FA concentration) for 15 min at 48°C. CARD signal amplification was done with 0.0015% H_2_O_2_ and Alexa488 tyramids (Invitrogen, Waltham, Massachusetts, United Kingdom) in amplification buffer (1x PBS, 2 M NaCl, 0.1% blocking reagent, 0.1 g ml^−1^ dextran sulfate). We used probes to target all bacteria (EUB I-III), SAR11 (SAR11-mix), *Bacteroidota* (CF319a), and *Gammaproteobacteria* (GAM42a, Table S1). After FISH, filters were embedded in citifluor: vectashield with DAPI, as described above, for microscopy.

### Microscopy and image analysis

Automated microscopy images were generated on a Zeiss AxioImager.Z2 m (Zeiss, Oberkochen, Germany) with a charged-coupled device (CCD) camera (Zeiss, Oberkochen, Germany) and a custom-built macro within the Zeiss AxioVision software (Zeder et al. 2011, Bennke et al. 2016). The microscope was equipped with a 63x Plan Apochromat objective (1.4 NA, oil immersion) objective. Samples were illuminated using a Zeiss Colibri 7 LED (excitation: 385 nm for DNA and 469 nm for 16S CARD-FISH) and multi-Zeiss 62 HE filter cube (Beam splitter FT 395+495+610). Recorded 8-bit greyscale images were manually quality controlled and analyzed in the automated cell measuring and enumeration tool (ACME, available from https://www.mpi-bremen.de/automated-microscopy.html) (Zeder et al. 2011, Bennke et al. 2016) with channel-specific settings (Table S2).

During the image analysis, we observed an unusual number of filamentous microbes, which were counted manually on a Zeiss AxioImager.D2, equipped with a Zeiss Colibri 7 LED. Filaments were quantified on a subset of samples (days 1, 2, 3, 7, 9, 11, 13, 15, 19, 23, and 27).

### DNA isolation and 16S rRNA gene sequencing

The prokaryotic community composition was assessed using 16S rRNA gene metabarcoding. Every Monday, Wednesday, and Friday, throughout the sampling campaign, a 500 ml sample from each mesocosm was sieved over a 150 μm nylon mesh and subsequently filtered through 3 μm and onto 0.2 μm pore-size polycarbonate filters (47 mm diameter, Millipore, USA). The filters were stored at −20°C until further processing. DNA was extracted using the NucleoSpin Soil extraction kit (Macherey-Nagel, Düren, Germany), according to the manufacturer’s instructions. An additional bead beating step was included for the lysis of the cells (MagNA Lyser, Roche, Switzerland). After DNA extraction, 5 ng µl^−1^ (final concentration) DNA was used for PCR amplification and library construction, according to Illumina’s 16S rRNA gene metabarcoding sequencing library preparation (https://support.illumina.com/downloads/16s_metagenomic_sequencing_library_preparation.html, doc.no:15044223B), with modifications for 16S rRNA gene amplicons preparation. The V4 region of the 16S rRNA gene was amplified using the forward MS_V4_515F_N (Parada et al. 2016) and reverse MS_V4_806R_1 (Apprill et al. 2015) primer (each 0.2 µM final concentration; Table S3) and the 2x KAPA HiFi HotStart ReadyMix (KAPABiosystems, Boston, USA). The PCR program included an initial denaturation at 95°C for 3 min, followed by 25 cycles of denaturation at 95°C for 30 s, annealing at 55°C for 30 s, extension at 72°C for 30 s, and a final extension at 72°C for 5 min. PCR products were cleaned with AMPure XP beads (BeckmanCoulter Life Sciences, Indianapolis, US), validated using the 2100 Bioanalyzer (Agilent), and pooled in equimolar concentrations. Amplicon libraries were sequenced on a MiSeq sequencer at the Alfred-Wegener-Institute, Bremerhaven, Germany, using the MiSeq Reagent Kit v3 (600-cycle) MS-102–3003. Retained sequences were trimmed with CUTADAPT 2.8 (Martin 2011). The DADA2 pipeline (Callahan et al. 2016) was used for quality control and defining amplicon sequence variants (ASVs). The taxonomical designation was done against the SILVA v138.1 Reference Database. The raw sequence data is available in the European Nucleotide Archive (Leinonen et al. 2010) under the accession number PRJEB89385, using the data brokerage service of the German Federation for Biological Data (Diepenbroek et al. 2014), in compliance with the Minimal Information about any (X) Sequence (MIxS) standard (Yilmaz et al. 2011).

### Statistical analysis and visualizations

Statistical analysis and visualizations were conducted in R v4.2.2 (R Core Team 2025) with the packages ggplot2 v3.4.2 (Wickham 2011), mgcv v1.8.42 (Wood 2011), cowplot v1.1.1 (Wilke 2020), and dplyr v1.1.2 (Wickham et al. 2023). All raw code is accessible on gitlab: https://gitlab.mpi-bremen.de/jbruewer/futureocean_microbes. We tested the influence of the heatwave and future coastal scenarios using generalized additive models on the following parameters: chlorophyll *a* concentrations, total cell counts, relative FISH abundances (for each FISH probe individually), Hill numbers, virus-like particle counts, cell division and grazing rates, and the number of counted filaments. In each case, the generalized additive models was tested with the formula *response variable ∼ s(Day of experiment, by=ERCP)+s(Day of experiment, by=Heatwave)+Heatwave+ERCP*, where *Heatwave* and *ERCP* were categorical (“yes” or “no”) and the smoothing term *Day of experiment* was provided in days. The effect of a particular treatment was tested using analysis of variance (ANOVA). Test results of the parametric terms are reported in this manuscript. In all cases, the smoothing terms were *p* < 0.05, indicating a significant contribution of explaining the variability in the response variable.

For downstream analysis of the 16S rRNA gene sequences, samples with in total less than 10 000 reads and ASVs with less than 10 reads were excluded from further analysis. Furthermore, ASVs not belonging to the domain Bacteria, related to mitochondria, or chloroplasts were removed prior further analysis. Next, counts per classification were normalized by calculating their relative abundances to the total number of SSU rRNA gene reads per sample. Permutational multivariate analysis of variance (PERMANOVA) was used to test for statistically significant variance of communities between the four different treatments. Two-way crossed design PERMANOVA was carried out with fixed factors and 9999 permutations at a significance level of *p* < 0.05. To visualize patterns of samples of the different treatments over time principal coordinates analysis (PCO) using Hellinger distance (D17) (Legendre and Legendre 1998) was performed. Hill numbers [(N1=EXP(H')] of the bacterial communities were calculated based on read counts of ASVs. Hill numbers, PERMANOVA, and PCO analysis were carried out with the Primer 7 software package plus the add-on package PERMANOVA+ (PRIMER-E Ltd, UK).

To determine genera that discriminated the four different scenarios from each other similarity percentage analysis (SIMPER) was applied. SIMPER was performed using Bray–Curtis similarity (S17) with fourth root transformed relative abundances.

For shade plot creation (Fig. S5) genera contributing most to the total dissimilarity (taxa of the first 10% of cumulative dissimilarity) between different scenarios were subjected to SIMPER analysis. Shade plot was created using square root transformed relative abundances and retaining the group of the sampling days. The taxa were sorted hierarchical based on variables standardized by total and the index of association as resemblance.

To test how single taxa respond to the different treatments we conducted additional MaAsLin (Microbiome Multivariable Association with Linear Models) analysis addressing the treatment affect (1) over all time points and (2) single time points. Multivariable association analysis and visualizations were conducted in R v4.5.2 (R Core Team 2025) with the packages Maaslin2 1.15.1 (Mallick et al. 2020) ggplot2 4.0.2 (Wickham 2011), and pheatmap_1.0.13 (Kolde 2015). Microbial log-transformed relative feature abundance was analyzed using linear-mixed effects models to evaluate the fixed effects of treatment, time and their interaction, with the random effect of mesocosm ID to account for measurements within the same mesocosm over time. The AMB treatment and the first sampling day served as reference level. To additionally assess each specific treatment-time combination, an additional model was fitted. In this model, a single categorical fixed effect was included, representing each treatment-sampling day combination. No random effects were included, as each treatment-sampling day combination represents a single observation per mesocosm. In this case, AMB day 1 (AMB_Day_1) served as reference level.

## Results

### Phytoplankton respond to future coastal scenario with little effect of heatwave treatments

**Figure 1:**
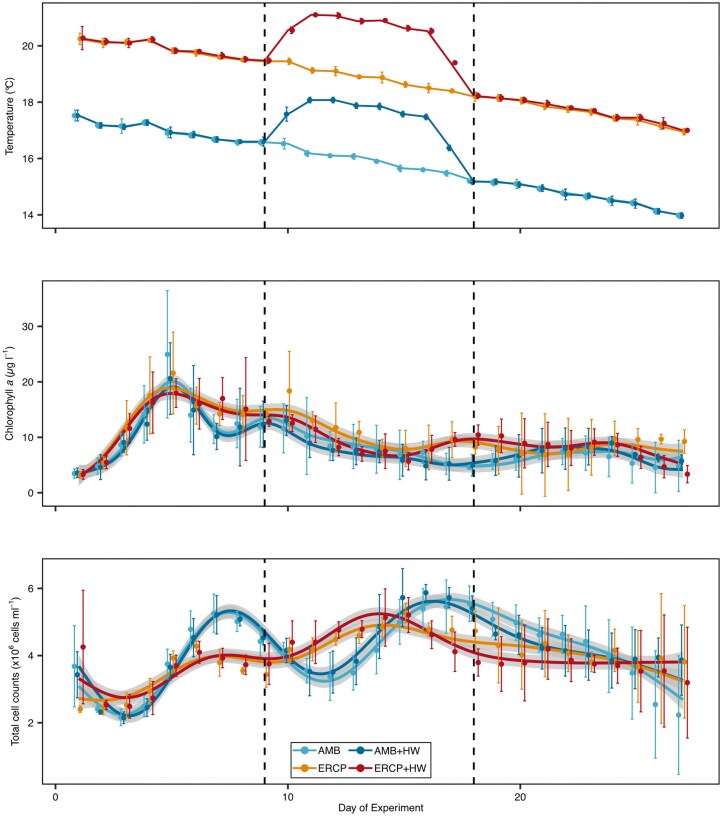
Temperature, chlorophyll *a* concentrations, and total cell counts during the sampling period. Vertical dashed lines indicate the beginning and end of heatwave treatment. Mean and standard deviation are shown across four replicates as dots and error bars, respectively. Colored curves are GAM models with 95% confidence intervals as ribbons. Temperature and total cell counts are based on Meunier et al. (2025).

Chlorophyll *a* concentrations, as a proxy for phytoplankton abundance, increased across all treatments from 3.4 ±0.4 µg l^−1^ (mean ±sd) to 21.3 ±4.3 µg l^−1^ within the first 5 days of the experiment (Fig. 1). Subsequently, concentrations decreased more or less gradually to starting concentrations until day 18 and 15 for the ambient (AMB & AMB+HW) and future coastal (ERCP & ERCP+HW) scenarios, respectively. Chlorophyll *a* concentrations peaked a second time on day ten (12.3 ±1.9 µg l^1^ in AMB & AMB+HW; 18.4 ±3.6 µg l^−1^ in ERCP), with the exception of the future coastal heatwave (12.6 ±0.8 µg l^−1^ in ERCP+HW) treatment. A likelihood ratio test of generalized additive models with and without the experimental treatments confirmed a significant effect of the experimental treatments (χ2=174.2, df=9.93, *P* < 0.001), although the effect of the MHW was primarily during the final two days of the experiment. Collectively, the applied generalized additive models explained 80.4% of the deviance.

### Future coastal scenarios and MHWs shape total cell counts

We measured total DAPI-stained cells cell counts using fluorescence microscopy. Total cell counts of the ambient (AMB & AMB+HW) and future coastal (ERCP & ERCP+HW) treatments showed significantly different temporal dynamics throughout the mesocosm experiments. A likelihood ratio test comparing generalized additive models with and without the future coastal scenario and MHWs identified a statistically significant effect of the experimental treatments (χ2=1.44 × 10^13^, df=8.89, *P* < 0.001), although the MHWs affected total cell counts to a lesser extent. The generalized additive model explained 80.1% of the deviance between predicted and observed values, indicating a strong model fit for the data.

In the ambient treatments, total cell counts initially decreased from 3.6 ±0.5 × 10^6^ cells ml^−1^ (mean ±sd) to 2.1 ± 0.1 × 10^6^ cells ml^−1^ within the first three days. Subsequently, the population increased to 5.2 ±0.3 × 10^6^ cells ml^−1^ (day seven), decreased to initial concentrations on day 12, increased a second time to reach 5.3 ±0.3 × 10^6^ (AMB) and 5.9 ±0.1 × 10^6^ (AMB+HW) cells ml^−1^ on day 16, and decreased thereafter. In contrast, total cell counts in the future coastal treatments (ERCP & ERCP+HW) were more stable and fluctuated less. They increased to 4.2 ±0.2 × 10^6^ cells ml^−1^ within the first six days, declined to 3.6 ±0.3 × 10^6^ cells ml^−1^ in the following three days and increased to 4.8 ±0.1 × 10^6^ (ERCP) and 5.1 ±0.4 × 10^6^ (ERCP+HW) cells ml^−1^ on day 15. Subsequently, total cell counts in these treatments declined slowly to reach starting concentrations by the end of the experiment. In both, the ambient and future coastal scenario, the MHW induced a minimally larger and earlier peak during the MHW, while cell counts were lower after the MHW (e.g., 4.3 ±0.6 (ERCP) and 3.7 ±0.4 (ERCP+HW) x10^6^ cells ml^−1^ on day 19).

Notably, as the experiment progressed, discrepancies between replicates of the same treatments became more pronounced after approximately three weeks, evident in the larger error bars (Fig. 1).

### Community specific effects in response to future coastal scenarios and heatwave exposure

**Figure 2:**
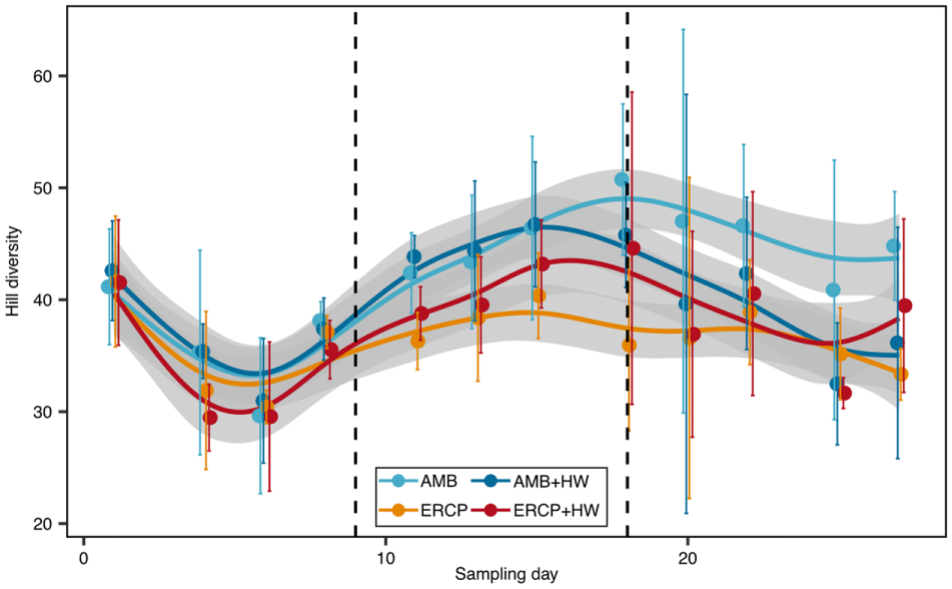
Diversity assessed through Hill numbers [N1=EXP(H')] based on observed ASVs. Colors represent experimental conditions. Dotted line indicates beginning and end of heatwave. Mean and standard deviation are shown across four replicates as dots and error bars, respectively. Colored curves are GAM models with 95% confidence intervals as ribbons.

We used 16S rRNA gene sequencing to investigate the differences of alpha and beta diversity of microbial communities between treatments and its changes over time during the experiment. The species diversity of the different samples, analyzed by Hill numbers [(N1=EXP(H')] based on observed ASVs, revealed significant differences between treatments and time (likelihood ratio test of generalized additive models; χ2=196.4, df=5.51, *p*=0.041; Fig. 2). Similar to the total cell counts, differences in Hill numbers were most prominent between the ambient and future coastal scenarios, with lower diversity values in the future coastal scenarios [42.2 ±1.8 (AMB), 43.8 ±0.9 (AMB+HW), 36.3 ±1.3 (ERCP), and 38.7 ±1.2 (ERCP+HW) on day 11; Fig. 2]. In the ambient conditions, the Hill diversity was lower, following the MHW [47.0 ±8.6 (AMB), 39.6 ±9.3 (AMB+HW) on day 20]. In contrast, in the future coastal scenario, the hill diversity was higher, following the MHW [35.9 ±3.8 (ERCP), 44.6 ±7.0 (ERCP+HW); Fig. 2].

**Figure 3:**
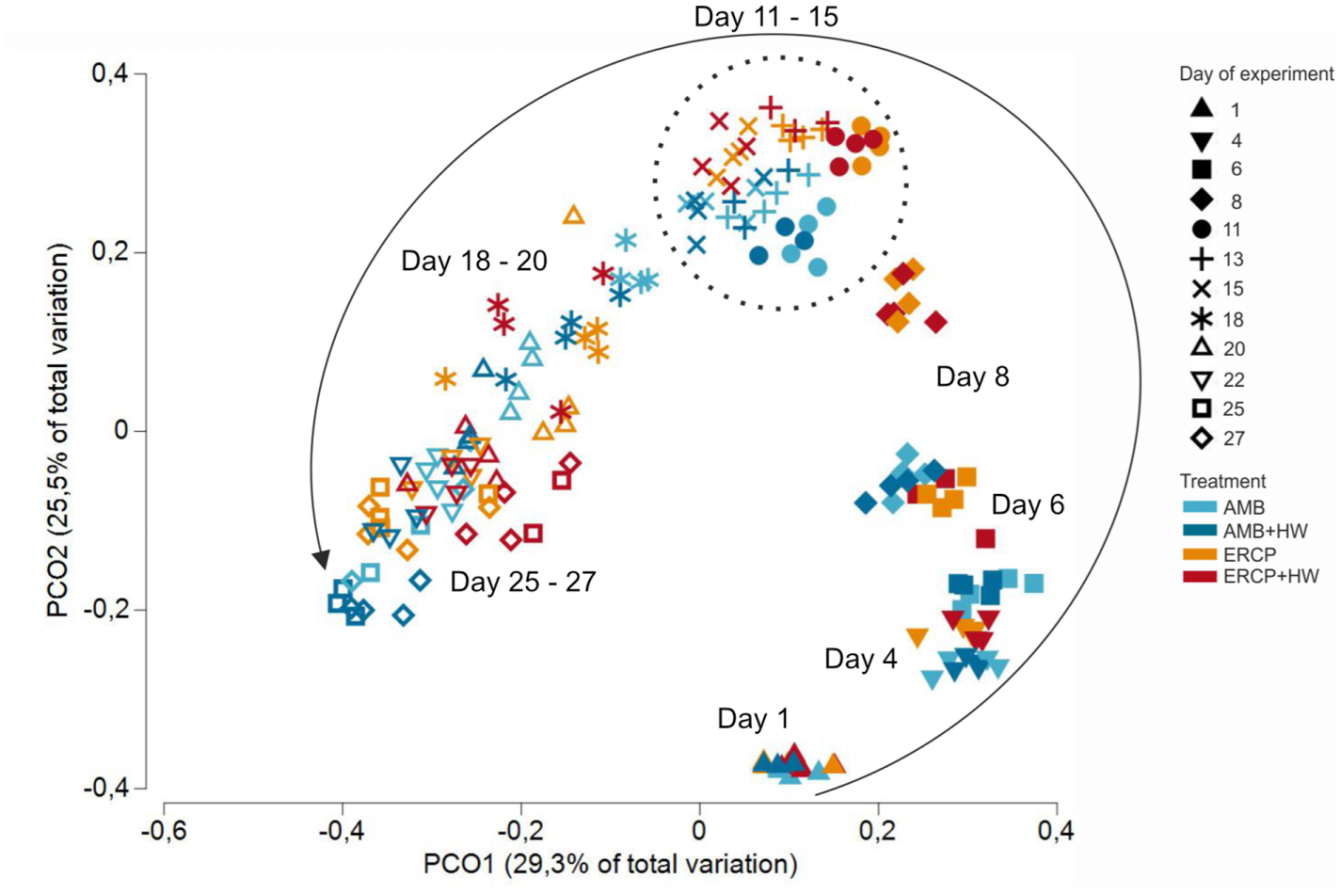
Principal Coordinate Ordination representing similarity of microbial communities based on relative abundances of ASVs across samples. Samples with less than 10 000 reads and ASVs with less than 10 reads were excluded. nAMB = 45; nAMB+HW = 45; nERCP = 48; nERCP+HW = 44 were analyzed. Symbols represent the day of the experiment. Colors represent experimental conditions. Dotted circle indicates communities during the heatwave. Circular arrow indicates the community development over time.

Principle coordinate analysis was used to visualize the similarities and dissimilarities between the communities in the different treatments (Fig. 3). First, all samples of all treatments in the beginning of the experiment cluster together and were clearly divided over time (day 1 and day 4, Fig. 3). On day 6 ambient and ERCP microbial communities split in bisection (Fig. 3). This splitting of ambient and ERCP communities can be observed until the end of the induced marine heatwave (day 15). Following to the marine heatwave the sample distribution in the multivariate space appears more heterogenous (Fig. 3). From PCO analysis we could not visually observe major effects on the microbial community composition of the applied heatwaves regardless of the treatment. However, the first two axes merely represent 54.8% of the total variation within the analyzed communities. To investigate if observed differences were significant, we performed a two-way crossed PERMANOVA including the factors “Day” and “Treatment” in an unbalanced design, whereas samples before the marine heatwave represent replicates of the ambient or ERCP treatments at a given time, respectively. We observed a statistically significant effect of the treatments and sampling day on bacterial communities with an interactive effect of time and treatment (PERMANOVA Day *P* = 0.0001; Treatment *P* = 0.0001; Day x Treatment *P* = 0.0001; Table S4). Pairwise comparison revealed main differences between the ambient (AMB) and future coastal (ERCP) treatments throughout the course of the experiment (*P* ≤ 0.05; Table S5). We observed significant effect on microbial communities following the MHW on day 18 in the AMB treatment (Table S5). However, no significant impact of the MHW could be observed between the ambient and future coastal treatments at any other time point.

**Figure 4:**
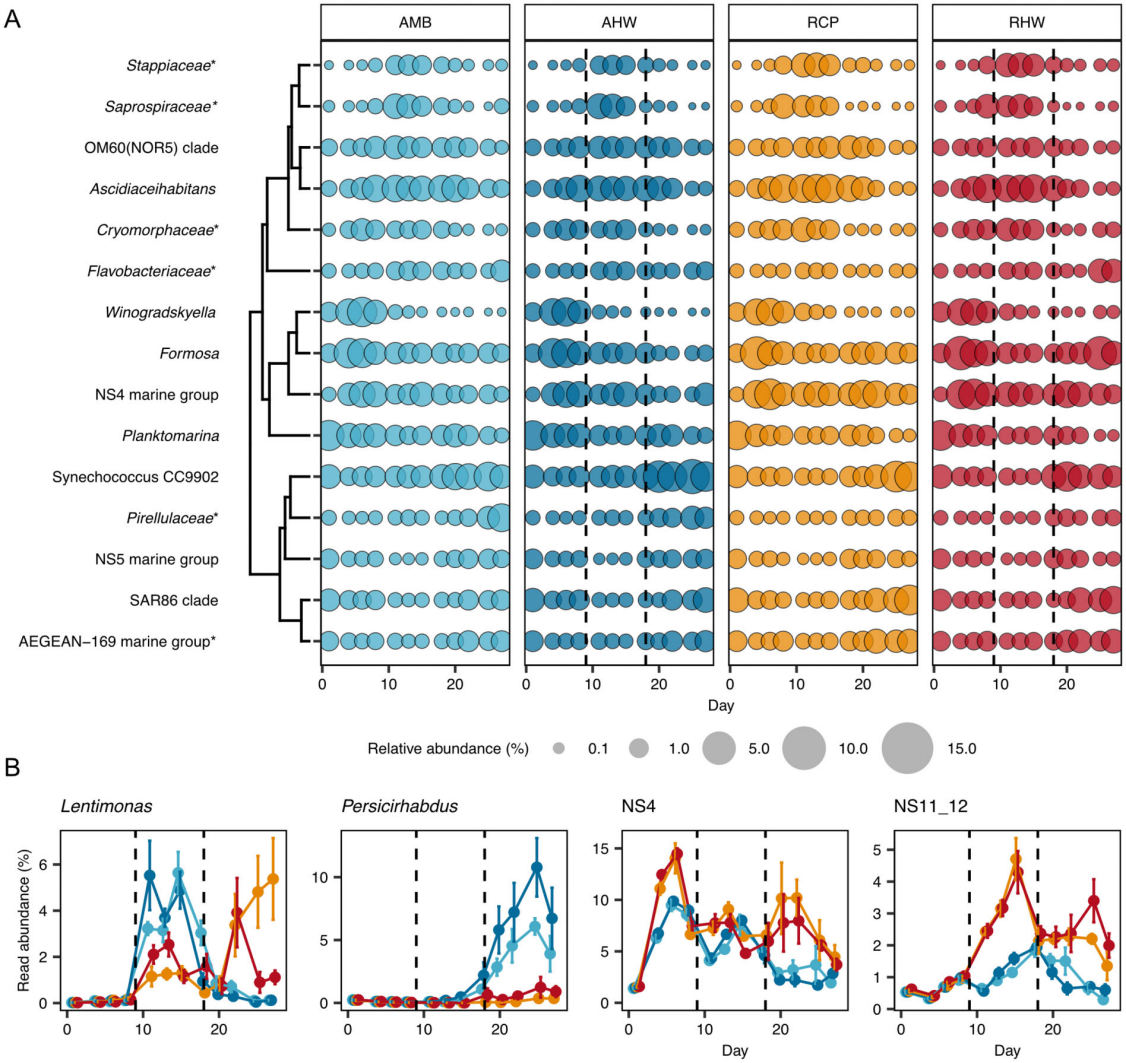
Microbial community composition assessed by 16S rRNA gene sequencing. (A) 15 most abundant genera. Mean of four replicates is indicated. (B) Selected individual taxa over the sampling campaign. Data represents mean with standard deviation (four replicates) as bars. Colors represent experimental conditions. Dotted line is beginning and end of heatwave. Asterisk indicate taxonomic groups identified on the family level only.

The alpha and beta diversity analysis of the 16S rRNA gene sequences indicated that the community composition changed over the course of the experiment and that MHWs had a lesser impact. Inspecting the data set from a taxonomic perspective, *Bacteroidota* (7.4%–59.5%), *Alphaproteobacteria* (13.5%–31.8%), and *Gammaproteobacteria* (9.2%–31.5%) contributed the most to 16S read abundances in all samples of all scenarios (Fig. 4 & S1–4 ). The top 15 most abundant groups reached the highest cumulative 16S rRNA gene read abundance on day 8 of the experiment in all treatments (Fig. 4). Overall, a SIMPER analysis revealed a change in the community composition over time and an average dissimilarity of 14%–16% between the different scenarios (ERCP & AMB 15.40%; AMB & AMB+HW 14.25%; ERCP & ERCP+HW 15.75%; Table S6). Additionally, SIMPER analysis revealed which taxa contributed most to the average dissimilarity between the different treatments (Table S5, Fig. S6 ), which included both, abundant and less abundant taxa. For example, *Synechococcus* and *Formosa* are both among the top 15 most abundant genera and the top 40 discriminating taxa. Both taxa increased in relative abundance following the MHW treatments in the ambient and future coastal scenarios, respectively (Fig. 4 & S5, Table S7). However, differences appeared even more prominent in less abundant taxa (Fig. S5 ).

To test how single taxa respond to the different treatments we conducted additional MaAsLin analysis addressing the treatment affect (1) over all time points (Fig. S6), and (2) single time points (Fig. S7), where AMB or AMB at the first day of experiment served as reference, respectively. First, in our model we observed significant results for the fixed effects “treatments” and “day of experiment” but not for their interaction. Since we are interested in the overall treatment effect, we excluded “day of experiment” for visualization of the results. Comparing AMB *vs* AMB+HW, ERCP, and ERCP+HW over all time points shows clearly, that major impacts on single taxa were observed in future scenarios, whereas taxa in AMB+HW showed no or slight response (Fig. S6). Inspecting single days of the experiment shows clearly the response of the “top 50” significant taxa over time in all treatments (Fig. S7). In general, we observed a cluster with negative response (lower cluster), positive response (upper cluster), and a mixture of negative and positive response (middle cluster) (Fig. S6, S7).

In the following, we selected examples to showcase taxon-specific response between the four scenarios. The 16S gene sequence read abundance of *Persicirhabdus* (*Verrucomicrobiota*) increased in the AMB treatment and even more so in the AMB+HW treatment, after 20 days but remained close to the detection limit in the future ocean scenarios. SIMPER analysis confirmed a high contribution of *Persicirhabdus* to the average dissimilarity between AMB and ERCP as well as AMB and AMB+HW scenarios. MaAsLin analysis revealed significant response of *Persicirhabdus* over the course of the experiment in both directions, respectively (Fig. S6 ). The *Bacteroidia* NS11–12 group appeared to benefit from the future coastal scenarios, while the effect of the applied heatwaves was limited (Fig. S7 ). NS4 marine group belonged to the highly discriminating taxa between ambient and future scenarios (Fig. S5; Table S6).

The 16S read abundance of *Lentimonas* (*Verrucomicrobiota*), increased in the ambient treatments after ca. 10 to 15 days and decreased to starting conditions thereafter. In the future coastal scenarios, the 16S gene sequence read abundance reached a peak after ca. 20 days. MaAsLin confirmed an overall positive response (or no response) in all treatments when inspecting single time points (Fig. S7). *Fluviicola* (*Bacterioda*) responded negatively to the future treatments (Fig. S7), whereas no significant change was observed in the AMB+HW treatment (Fig. S6).

To complement 16S-gene-sequencing-derived abundances, we determined the abundances of SAR11, *Gammaproteobacteria, Bacteroidia*, and all bacteria (probe: EUB338 I-III) using fluorescence *in situ* hybridization in a single replicate (Fig. S8). Different to the 16S analysis, the microscopy-based analysis revealed that the community composition consisted of >20% SAR11 cells (Fig. S8), which can be explained by known primer biases of 16S gene sequencing approaches (Lee et al. 2023). *Bacteroidota* and *Gammaproteobacteria* contributed less than 25 and 10% to the communities (Fig. S8, Table S6), respectively.

### Dilution experiments indicate differing effects of MHW on cell division and grazing rates

**Figure 5:**
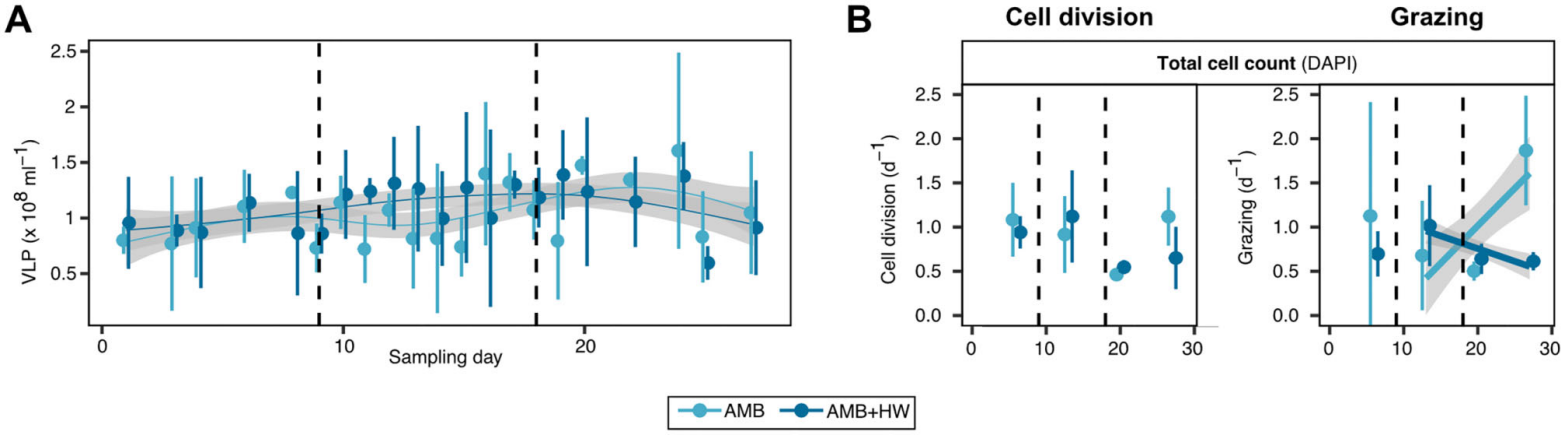
Microbial mortality and cell division rates. (A) Virus-like particle concentrations. Shown are mean and standard deviation across four replicates as dots and error bars, respectively. Colored curves are generalized additive models with 95% confidence intervals as ribbons. (B) Microscopically determined cell division and grazing rates for all bacteria (assessed by DAPI). Shown are mean with standard deviations (four replicates). Data for individual taxonomic groups can be found in the supplements (Fig. S8). Dashed lines indicate beginning and end of the heatwave treatment.

To assess the influence of a MHW on cell division and grazing rates in ambient conditions, we conducted dilution experiments once a week. First, we assessed cell division and grazing for total cell counts (i.e., all microbial cells identified with DAPI). Over all samples and replicates, cell division rates 0.9 ±0.3 d^−1^ (mean ±sd) were comparable to grazing rates 0.9 ±0.5 d^−1^ (Fig. 5). We could not detect an influence of the heatwave on cell division rates based on total cell counts (F(1) =0.734, *p* = 0.40), while grazing rates decreased after the MHW, compared to the control (F(1)=9.38, *p* = 0.006).

Additionally, we investigated taxon-specific cell division and grazing rates on SAR11 (n=4), *Bacteroidota* (n=1), and *Gammaproteobacteria* (n=1). SAR11 cell division rates averaged 0.6 ±0.3 d^−1^ and were stable over time (Fig. S8). We could not detect an effect of the MHW on cell division (F(1)=3.214, *p* = 0.091) nor grazing rates (F(1)=0.042, *p* = 0.84; Fig. S8). However, grazing decreased from 0.7 ±0.3 d^−1^ on day six to 0.2 ±0.3 d^−1^ on day 13. Subsequently, grazing rates increased again towards starting conditions (Fig. S8). Besides SAR11, we got first insights into cell division and grazing rates of *Bacteroidota* and *Gammaproteobacteria* (Fig. S8). Cell division rates were faster than those of SAR11 and the average of all bacteria (*Bacteroidota*: 1.1 ±0.9; *Gammaproteobacteria*: 1.4 ±0.7 division d^−1^). Similarly, grazing rates were higher and comparable to cell division rates (*Bacteroidota*: 1.4 ±0.9; *Gammaproteobacteria*: 1.4 ±1.2 d^−1^). Both, cell division and grazing rates, were not affected by the MHW (no statistics applied; Fig. S8).

During the sample preparation of the total cell counts, we noticed considerable amounts of filamentous microbial cells. Filaments were absent in the beginning of the incubation, increased to 1.0 × 10^5^ ±0.1 × 10^5^ (mean ±sd) filaments ml^−1^ and 1.8 × 10^5^ ±0.1 × 10^5^ filaments ml^−1^ on day 14 and 12 for the ambient (AMB & AMB+HW) and future coastal (ERCP & ERCP+HW) treatments, respectively (Fig. S9). Filaments were more abundant and peaked earlier in the future coastal scenarios (F(1)=109.6, *p* < 0.001), however, an effect of the MHW could not be detected (likelihood ratio test, χ2=1.2 × 10^8^, df=3.0, *p* = 0.90). The generalized additive model explained 94.4% of the deviance, indicating a good representation of the data.

### No indications of heatwave impact on virus-like particle counts

To assess the impact of a MHW on the viral community in ambient condition, we additionally studied virus-like particles (VLP) using fluorescence microscopy. Concentrations were more than 10-fold higher than total cell counts (Fig. 5). Their fluctuations over the sampling period were less than those of total cell counts, while the variance between replicates was much greater. VLP concentrations increased from 8.8 ±1.6 × 10^7^ VLP ml^−1^ to 13.5 ±2.5 × 10^7^ VLP ml^−1^ after 20 days (Fig. 5). This increase was steady for the heatwave replicates, whereas a minor decrease in abundances could be observed in the ambient control treatment. However, no significant influence could be detected of the MHW on the VLP counts (F(1)=2.411, *P* = 0.123), suggesting a limited impact of the applied heatwave on virus-induced lysis.

## Discussion

Anthropogenic influences are shaping the future coastal ocean but to what extent is the microbial community affected? We assessed the impacts of future coastal conditions (increased CO_2_, temperature, and N: P ratio), as well as the exposure to a marine heatwave, on the microbial community. Our results indicate an influence of future coastal scenarios on microbial cell counts, biodiversity, and community composition. In contrast, the MHW had no effect on the cell division and virus-like particle counts, while minor negative effects could be observed on grazing rates. Generally, our results indicate a rather resilient microbial community to short-term moderate MHWs.

### Effect of future coastal scenario on microbial communities

The bacterial community reacted to the future coastal scenario with an earlier second bloom in cell abundances and less fluctuation overall, most likely due to bottom-up (substrate-driven) processes. It is known that phytoplankton increase their photosynthetic activity in response to elevated temperatures and CO_2_ levels (Gattuso et al. 2015). Moreover, the composition of carbohydrates in organic matter derived from phytoplankton has been observed to vary depending on environmental conditions (Mühlenbruch et al. 2018) and during phytoplankton blooms (Teeling et al. 2012, Vidal-Melgosa et al. 2021). Effects on phytoplankton biomass (Meunier et al. 2025) and Chlorophyll *a* (this study) were close to negligible. However, the phototrophic community composition—assessed by 18S rRNA gene sequencing—was affected by the future coastal scenarios (Ahme et al. 2025), indicating that the substrate availability and composition was different in these samples. Hence, we hypothesize that observed changes in cell counts and community compositions were, at least partly, substrate driven.

Generally, environmental microbial communities are shaped by bottom-up and top-down (i.e. mortality by grazing or virus-induced lysis) processes. The occurrence of filament formation in the present study might indicate high grazing pressure (Jürgens et al. 1999, Pernthaler et al. 2004), which would be supported by the finding of rapidly increasing grazer abundances across treatments, reported in (Meunier et al. 2025). However, filaments were more abundant and occurred earlier in the future coastal scenarios, although grazers were more abundant in the ambient treatments (Meunier et al. 2025). Whether this conundrum is due to altered grazing efficiencies or increased stress response in the future ocean scenario, remains subject to future studies.

The effect of the future coastal scenario was also apparent on a community composition and taxon-specific level. The most pronounced differences in 16S rRNA gene read abundances could be observed in less abundant taxonomic groups, which could hint towards altered ecological conditions of the future coastal scenario. For example, *Lentimonas* is specialized in digesting the highly complex sulfated polysaccharide fucoidan (Sichert et al. 2020), which is produced by e.g. brown algae. Higher abundances of *Lentimonas* in the future coastal treatments might hint toward increased amounts of fucoidan after ca. 15 days. This aligns with higher relative abundances of brown algae-parasites in these mesocosms (Ahme et al. 2025), highlighting a potential role of brown-algae and corresponding exudates in future coastal conditions.

Several bacterial taxa commonly associated with phytoplankton blooms (NS11-12 & NS4 marine group) (Meziti et al. 2015, Xu et al. 2021) are more abundant in the future coastal scenarios (this study). Likewise, (Meunier et al. 2025) reported higher abundances of potentially pathogenic *Vibrio* bacteria in the future coastal scenarios. In contrast, other taxa commonly associated with phytoplankton blooms (e.g. *Formosa, Flavobacteria*, amongst others) and well-studied in the North Sea (Teeling et al. 2016, Sidhu et al. 2023) do not depict differential abundances or are even less abundant in the future coastal treatments, which might be due to altered substrate availability, as discussed above. In summary, we observed minor changes in the bacterial community composition and cell abundances in response to the future coastal scenario, which likely result from altered bottom-up effects.

### The microbial community is largely resistant to marine heatwaves

The microbial community appeared to be well able to cope with the short-term MHW. Besides an earlier second bacterial bloom, the MaAsLin analysis revealed that the community composition changed to a lesser extent or not at all, despite the short-term marine heatwave. In contrast, previously observed effects on the microbial community were a result of much longer elevated temperatures [or multiple MHW in the time-span of a few months (Brown et al. 2024)]. We expected to observe increased microbial productivity and cell division rates during heatwaves (Hutchins and Fu 2017), which might in turn be compensated by higher mortality rates (Moustaka-Gouni et al. 2016, Machado et al. 2020). However, in the presented study, the heatwave treatments did not have any noticeable effect on the cell division rates, while grazing rates were higher in the control than in the MHW treatment after >1 week following the MHW. This difference might either be explained by higher observed ciliate abundances at the same time-point [reported in (Meunier et al. 2025)] or indicate an impact of the short-term temperature increase on the diet of the grazers. However, no differences could be observed for taxon-specific grazing or cell division rates of SAR11, *Bacteroidota*, or *Gammaproteobacteria*.

Besides mortality through grazing, we also assessed VLP counts in all ambient treatments. We hypothesized that rising temperatures and the potential stress from the heatwave would result in increased VLP counts, e.g. by triggering prophages to enter the lytic cycle (Breitbart 2012, but see also: Brüwer et al. 2024). However, the observed increase in the MHW treatment was statistically not significant. This suggests that VLP counts, similar to the host abundances, is resilient to the moderate MHW.

Our experimental design aimed to assess the influences of marine heatwaves on the microbial community in ambient and future ocean scenarios. While the temperature for the simulated marine heatwaves was raised over two days to slowly increase the temperature, the conditions for the future ocean treatments (+3°C, 1000 ppm CO_2_, and N: P ratio of 25) were applied within much shorter timeframes. The future ocean will change over decades, meaning that the microbiome can adapt to the new conditions slowly. Considering short generation times of microorganisms, the microbial community is able to evolve and adapt quickly (Hutchins and Fu 2017). Additionally, the high abundance of filaments could be due to limited water movement, which have been observed in mesocosm studies previously (Riemann et al. 2000). Despite those recognized limitations, mesocosms are valuable for their ability to capture the full breadth of species and genetic diversity, while offering the flexibility for controlled experimental manipulations (Moustaka-Gouni et al. 2016).

To summarize, our study shows that while heatwaves do not significantly impact planktonic microbes, simultaneous changes in temperature, pCO2, and dissolved N: P ratios significantly alter the microbial diversity, community composition, and cell abundances. Further, our results suggest that observed shifts in the bacterial community are, at least partly, shaped by bottom-up processes, i.e. changes in substrate availability from primary producers. This shift might have broader implications on biogeochemical cycles, which is subject to future studies. In contrast, the tested influences of short-term temperature increase during a marine heatwave indicate a rather resilient microbial community. While we observed an earlier bacterial bloom, the overall microbial community remained relatively stable. Overall, our results highlight the capacity of marine microbial communities to cope with heatwaves but also raise questions about long-term shifts in response to ongoing oceanic changes.

## Supplementary Material

fiag042_Supplemental_Files
