# Supplementary material for: Resilience and response of marine microbes to the future ocean and a marine heatwave — insights from a mesocosm experiment
Source: FEMS Microbiol Ecol. 2026 Apr 22;102(5):fiag042. doi: 10.1093/femsec/fiag042 (PMC13152010; doi:10.1093/femsec/fiag042)
Supplement: fiag042_Supplemental_Files [file fiag042_supplemental_files.zip › 05_Supplement_r1.docx]

# Supplement

# Response and resilience of marine microbes to the future ocean and a marine heatwave – insights from a mesocosm experiment

Jan D. Brüwer^1*^, Micah Reismann^1^, Antje Wichels^2^, Uwe John^2^, Josefin Schmidt^2^, Cédric L. Meunier^2^, Bernhard M. Fuchs^1^, Inga V. Kirstein^2^

^1^ Max Planck Institute for Marine Microbiology, Bremen, Germany

^2^ Alfred-Wegener-Institut, Helmholtz-Zentrum für Polar- und Meeresforschung, Biologische Anstalt Helgoland, Helgoland, Germany

*Corresponding author: jbruewer@mpi-bremen.de; bruewer_j@gmx.de

**Supplementary Figures**


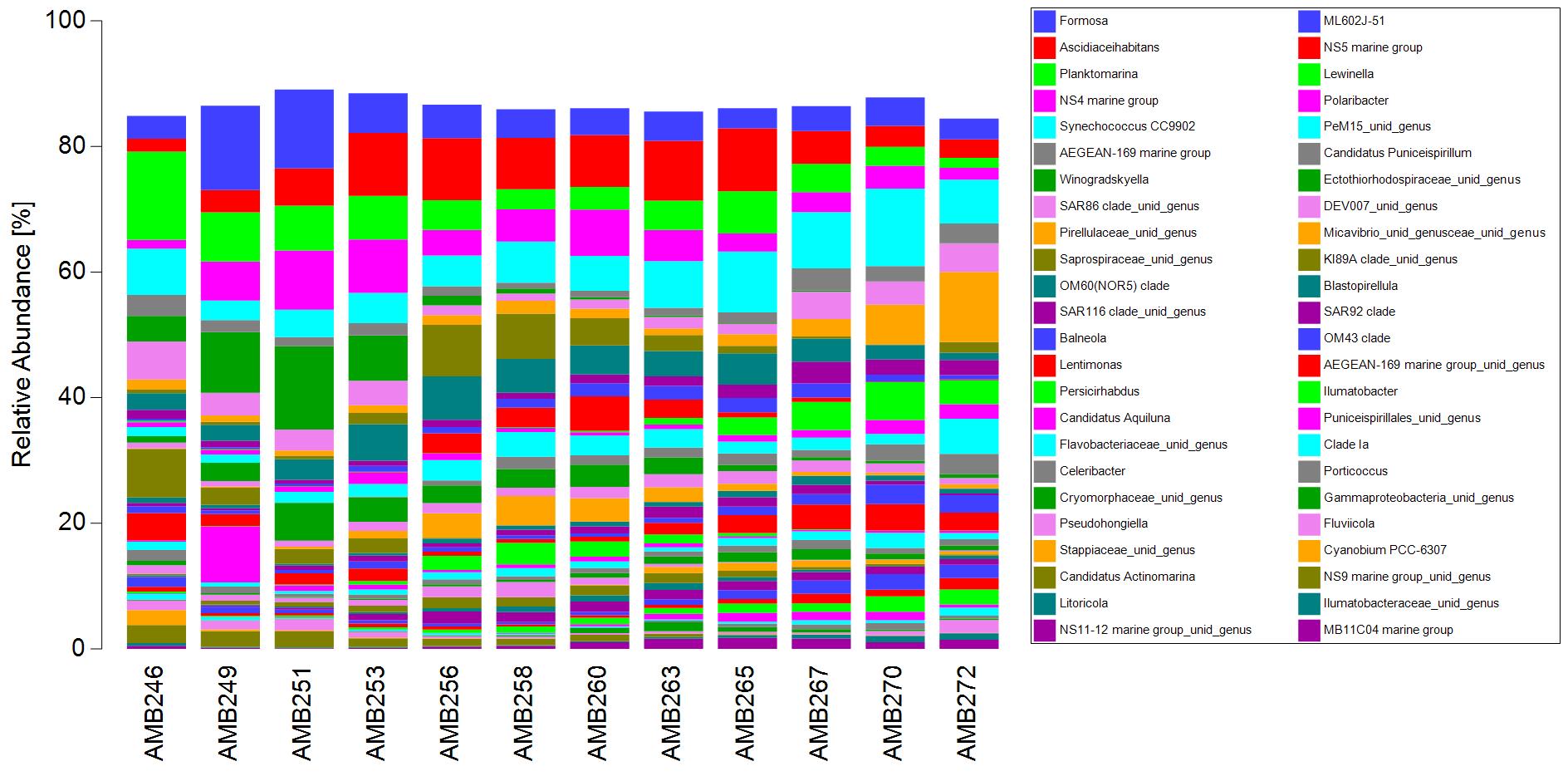
**Figure S1: Abundance profiles of bacterial genera in ambient (AMB)** treatments at different sampling day indicated as Julian day. Displayed are taxonomic genera contributing to at least 1%.


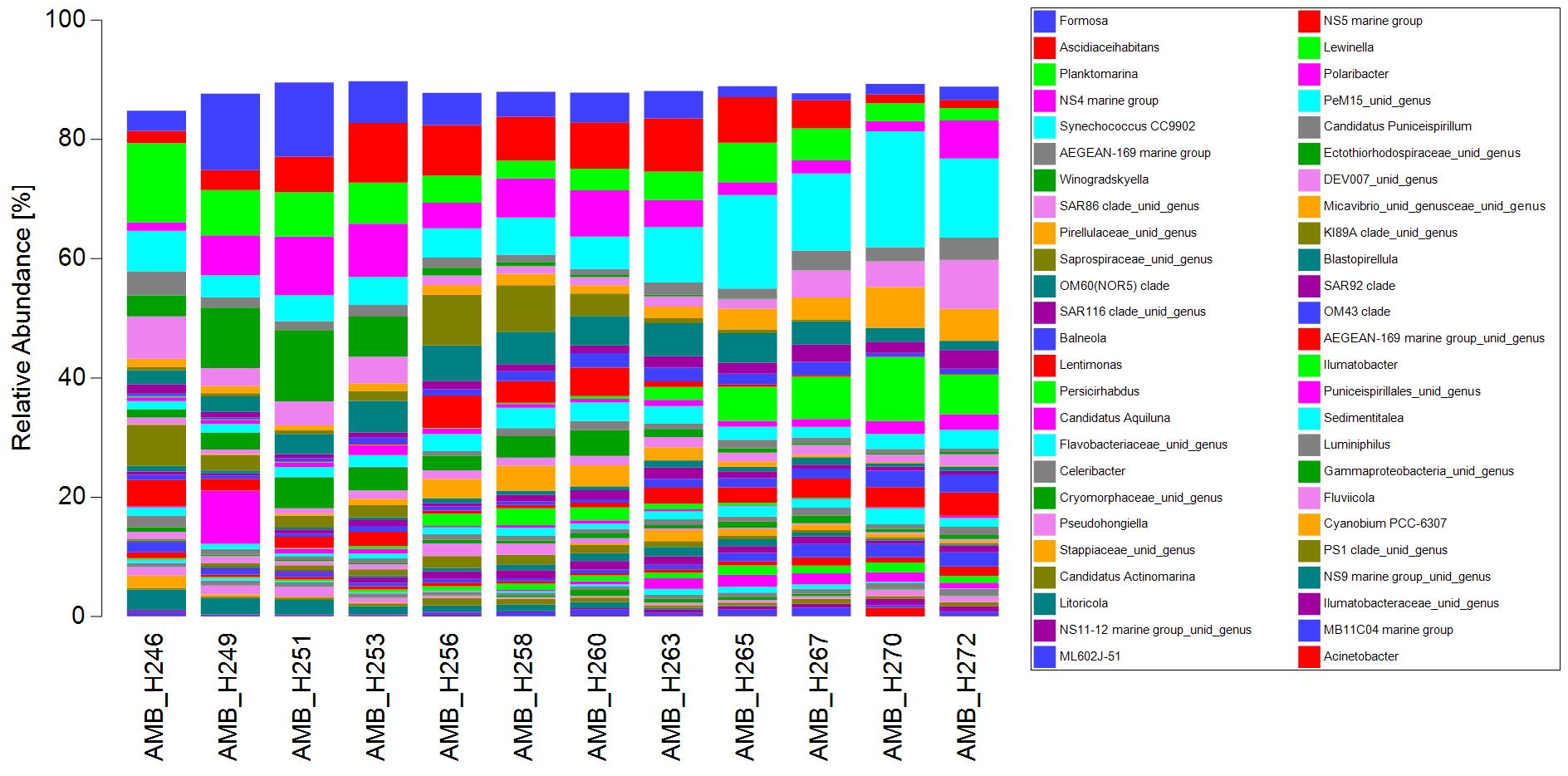
**Figure S2: Abundance profiles of bacterial genera in ambient + heatwave (AMB+HW)** treatments at different sampling day indicated as Julian day. Displayed are taxonomic genera contributing to at least 1%.


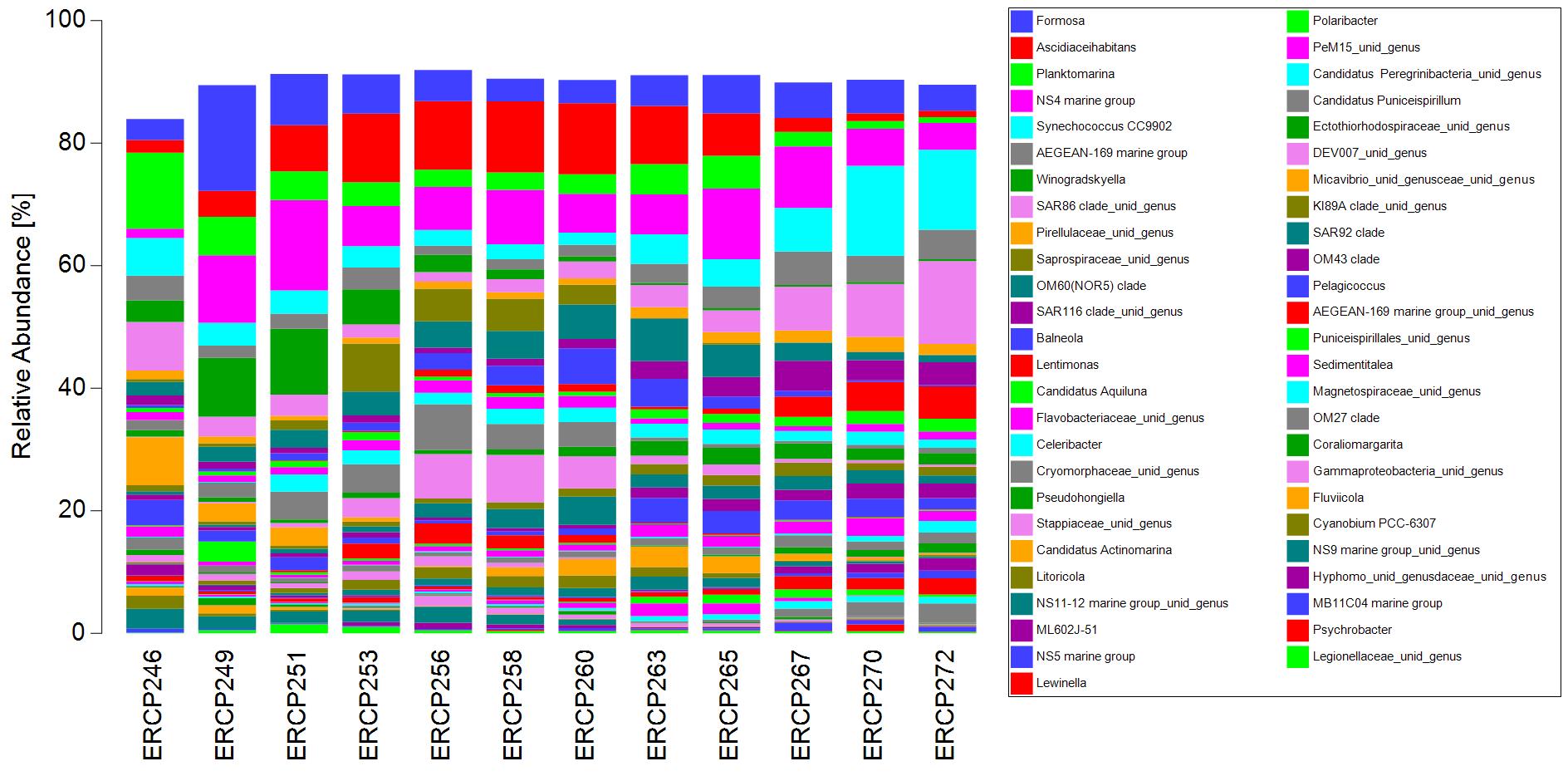
 **Figure S3: Abundance profiles of bacterial genera in extended RCP (ERCP)** treatments at different sampling day indicated as Julian day. Displayed are taxonomic genera contributing to at least 1%.


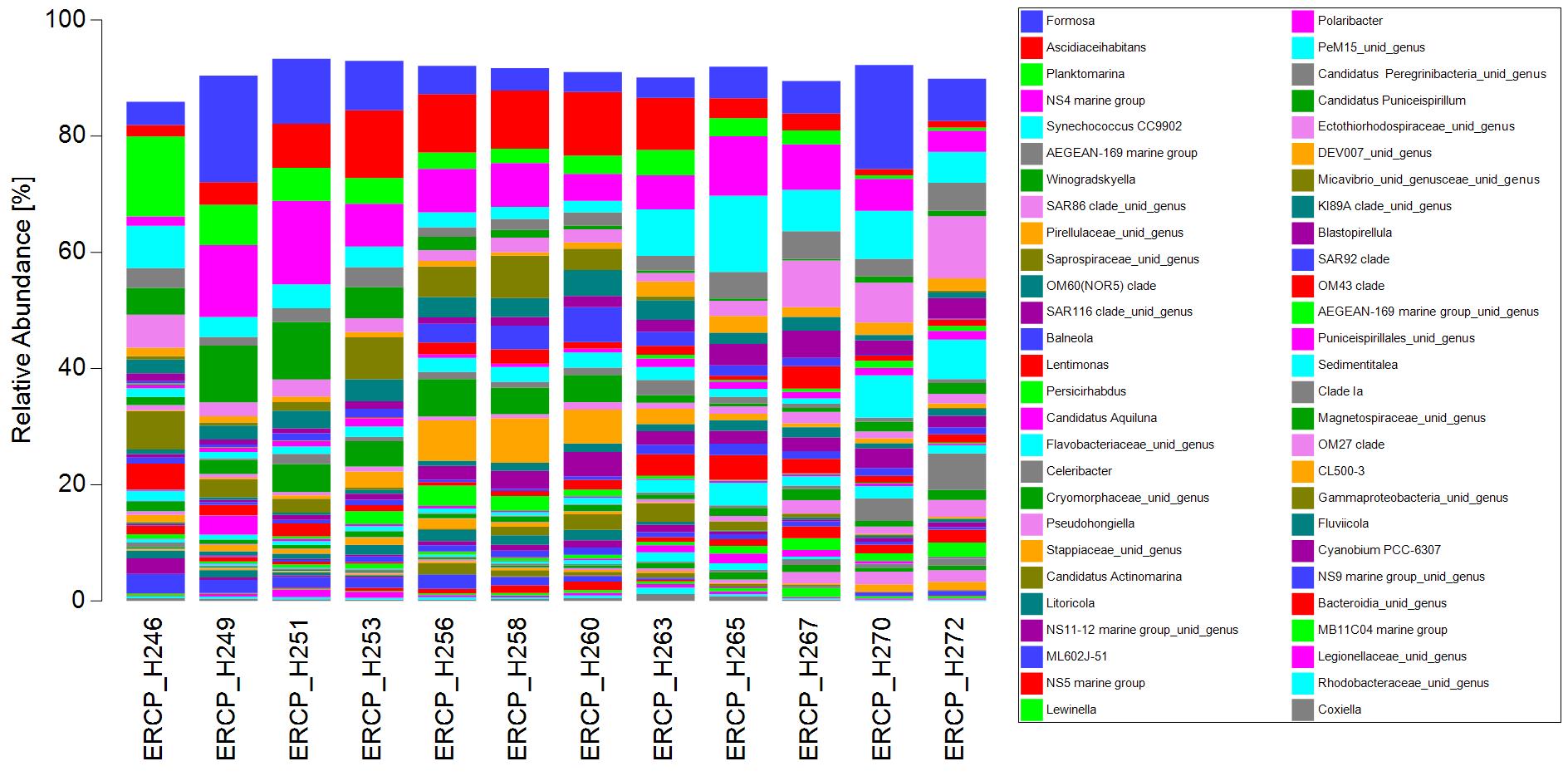
 **Figure S4: Abundance profiles of bacterial genera in extended RCP + heatwave (ERCP+HW)** treatments at different sampling day indicated as Julian day. Displayed are taxonomic genera contributing to at least 1%.


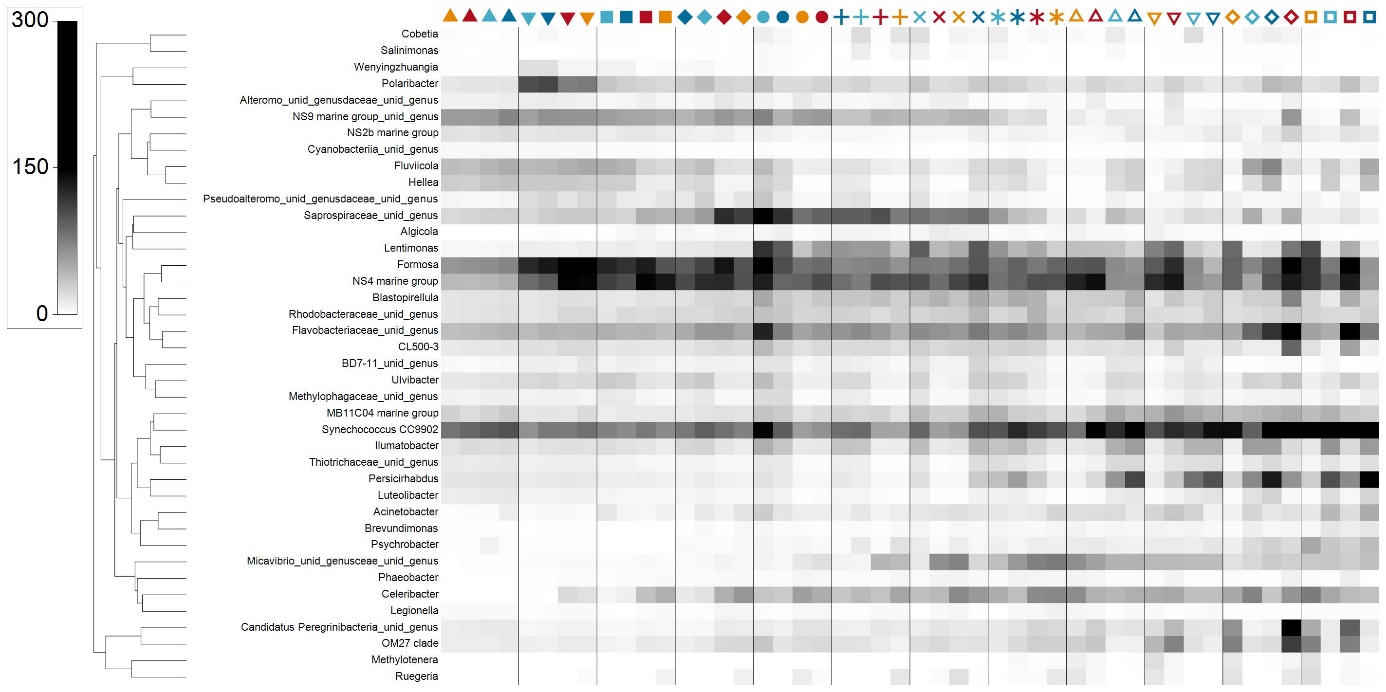


**Figure S5: Shade Plot of top 40 dissimilar taxa.** Shade Plot creation was based on standardized, square root transformed mean values of the respective treatment and day (in total: nAMB = 45; nAMB+HW = 45; nERCP = 48; nERCP+HW = 44). Displayed are taxa on the genus level highly contributing to the first 10% of the cumulative dissimilarity between different scenarios (SIMPER analysis).


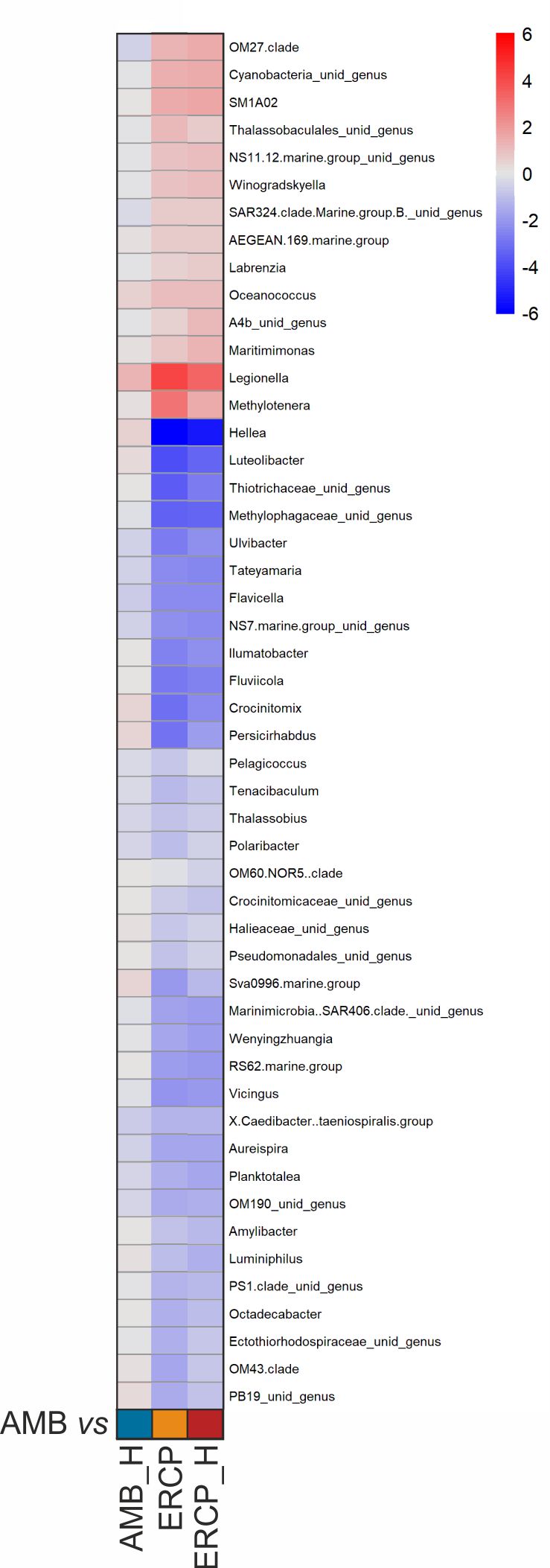


**Figure S6: MaAsLin analysis of treatment effect over all time-points.** Top 50 taxonomic groups, contributing the most to changes in the microbial community are displayed. Each treatment was compared to the Ambient treatment. MaAsLin is based on log transformed relative abundances.


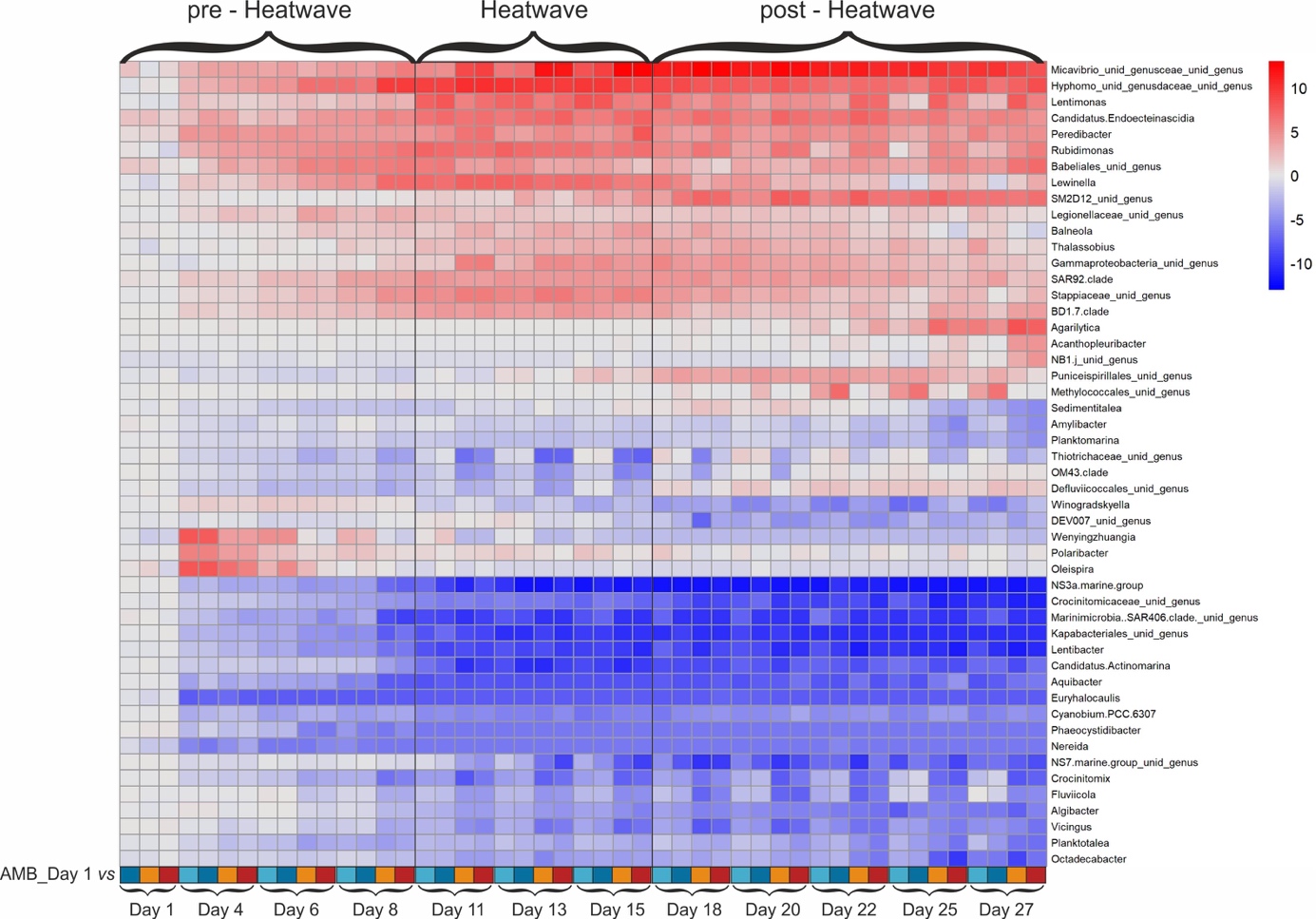


**Figure S7: MaAsLin analysis of treatment effect for each day.** Top 50 taxonomic groups, contributing the most to changes in the microbial community are displayed. Each treatment at each timepoint was compared against the ambient treatment at day 1. MaAsLin is based on log transformed relative abundances.


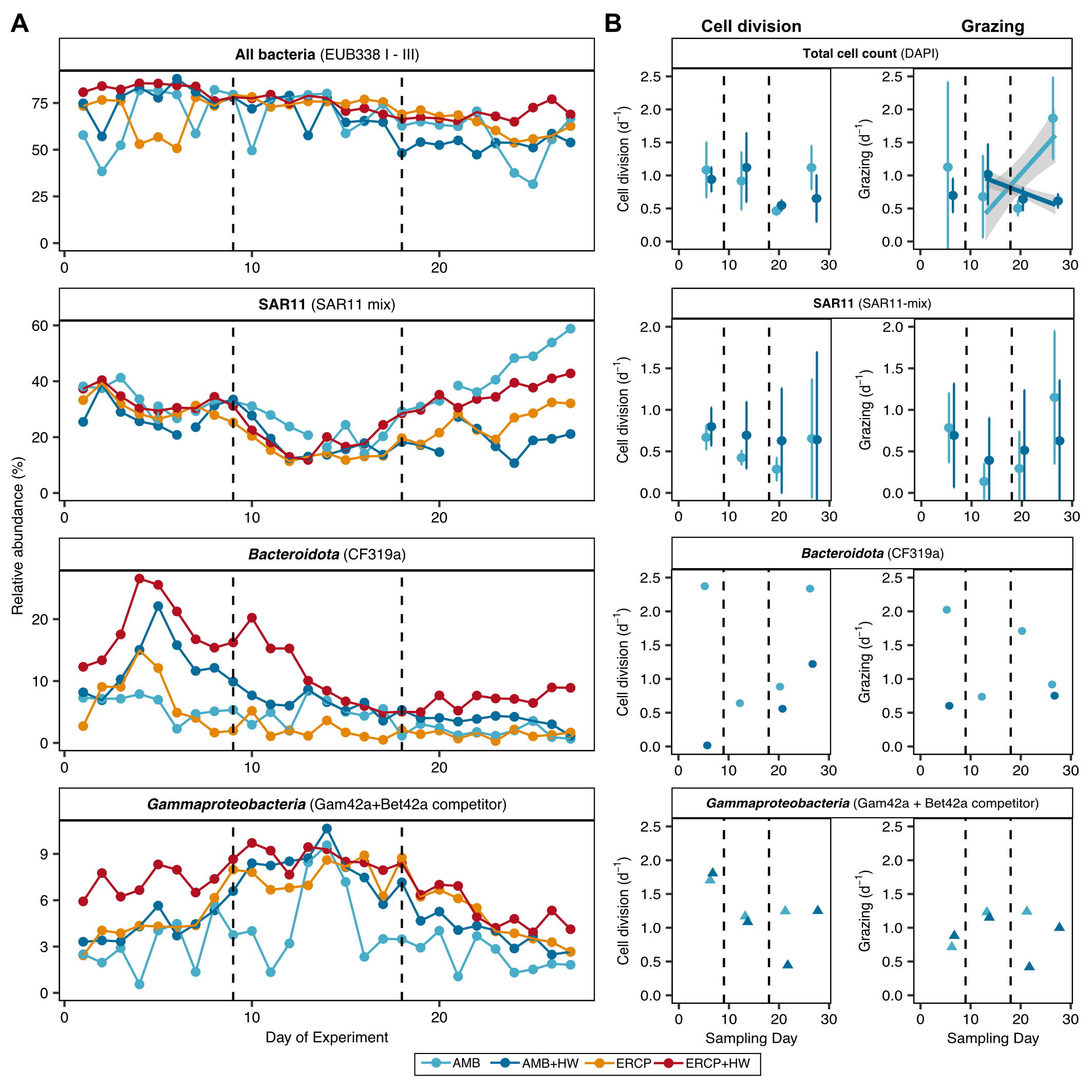


**Figure S8: Microscopically determined abundances and life cycle measures (cell division and grazing).** (A) Cell abundances determined by FISH (n=1). (B) Experimentally determined cell division and grazing rates. Plot for total cell counts (DAPI) is a replica of the main manuscript. Dots show mean with standard deviation (n=4) as bars. Dashed lines indicate beginning and end of the heatwave treatment.


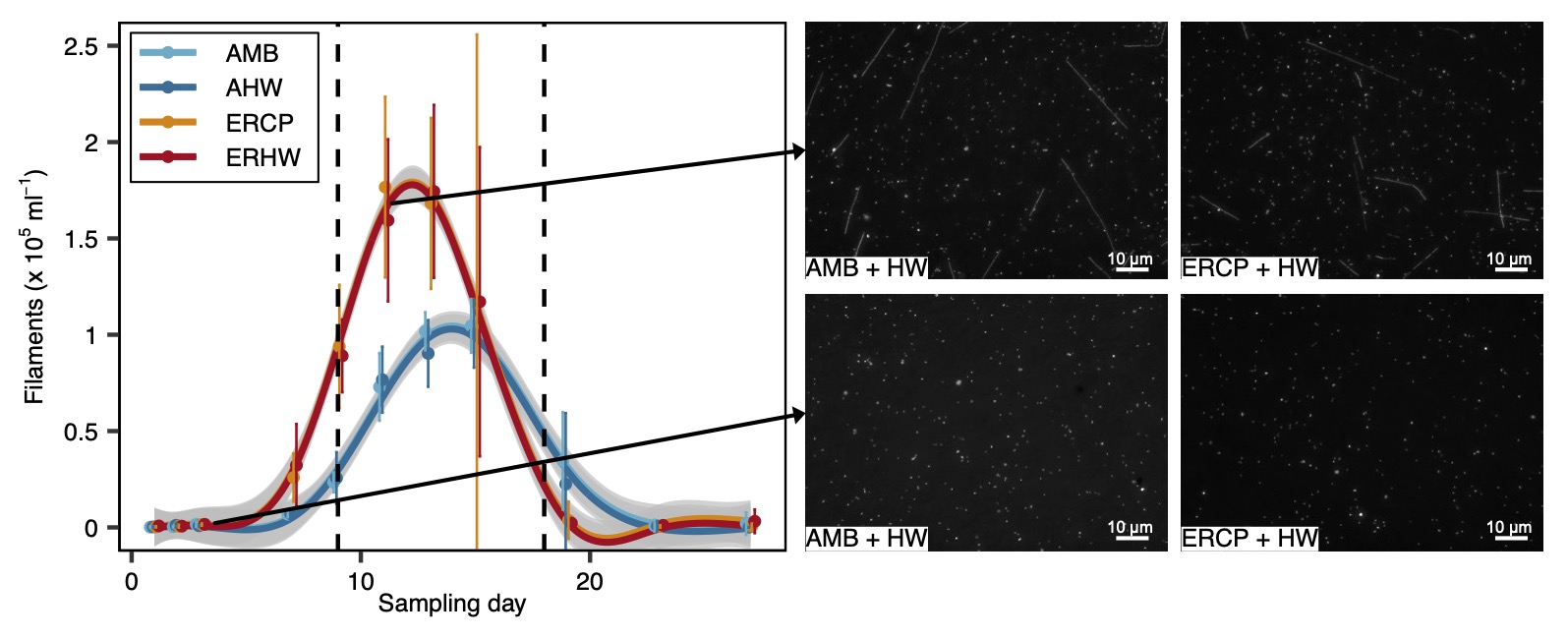


**Figure S9: Abundance of filamentous bacteria** during sampling period. Dashed lines indicate beginning of heatwave treatment. Shown are mean and standard deviation across four replicates as dots and error bars, respectively. Colored curves are GAM models with 95% confidence intervals as ribbons. Microscopy images were recorded automatically (see methods), scale bars were added manually.
